# Supplementary material for: In the context of romantic attraction, beautification can increase assertiveness in women
Source: PLoS One. 2020 Mar 10;15(3):e0229162. doi: 10.1371/journal.pone.0229162 (PMC7064170; doi:10.1371/journal.pone.0229162)
Supplement: S1 Appendix — (DOCX) [file pone.0229162.s001.docx]

**Table A. Experiment 1, Correlations between variables.**

|  | | Implicit assertiveness | Explicit assertiveness | Behavioral assertiveness | Sexual motivation | Positive affect |
| --- | --- | --- | --- | --- | --- | --- |
| Implicit assertiveness | Pearson R | 1 |  |  |  |  |
|  | Sig. |  |  |  |  |  |
|  | N | 142 |  |  |  |  |
| Explicit assertiveness | Pearson R | .140 | 1 |  |  |  |
|  | Sig. | .095 |  |  |  |  |
|  | N | 142 | 145 |  |  |  |
| Behavioral assertiveness | Pearson R | .179 | .290 | 1 |  |  |
|  | Sig. | .164 | .021 |  |  |  |
|  | N | 62 | 63 | 63 |  |  |
| Sexual motivation | Pearson R | .040 | .299 | .297 | 1 |  |
|  | Sig. | .636 | .000 | .018 |  |  |
|  | N | 142 | 145 | 63 | 145 |  |
| Positive affect | Pearson R | .134 | .187 | .139 | .368 | 1 |
|  | Sig. | .113 | .025 | .276 | .000 |  |
|  | N | 142 | 145 | 63 | 145 | 145 |
| *Note.* *. Correlation is significant at the 0.05 level (2-tailed). **. Correlation is significant at the 0.01 level (2-tailed). | | | | | | |
|  | | | | | | |

**Table B. Experiment 2, Correlations between variables**

|  | | Baseline explicit assertiveness | Experimental explicit assertiveness | Baseline vignette 1 | Baseline vignette 2 | Baseline vignette 3 | Experimental vignette 1 | Experimental vignette 2 | Experimental vignette 3 | Sexual motivation (Experimental) |
| --- | --- | --- | --- | --- | --- | --- | --- | --- | --- | --- |
| Experimental explicit assertiveness | Pearson R | .631^**^ |  |  |  |  |  |  |  |  |
|  | Sig. | .000 |  |  |  |  |  |  |  |  |
|  | N | 40 | 40 |  |  |  |  |  |  |  |
| Baseline vignette 1 | Pearson R | .278 | .363^*^ |  |  |  |  |  |  |  |
|  | Sig. | .082 | .022 |  |  |  |  |  |  |  |
|  | N | 40 | 40 | 40 |  |  |  |  |  |  |
| Baseline vignette 2 | Pearson R | -.018 | -.076 | .334^*^ |  |  |  |  |  |  |
|  | Sig. | .911 | .643 | .035 |  |  |  |  |  |  |
|  | N | 40 | 40 | 40 | 40 |  |  |  |  |  |
| Baseline vignette 3 | Pearson R | .292 | .222 | .309 | .231 |  |  |  |  |  |
|  | Sig. | .067 | .168 | .053 | .151 |  |  |  |  |  |
|  | N | 40 | 40 | 40 | 40 | 40 |  |  |  |  |
| Experimental vignette 1 | Pearson R | .263 | .215 | .675^**^ | .216 | .276 |  |  |  |  |
|  | Sig. | .102 | .182 | .000 | .181 | .084 |  |  |  |  |
|  | N | 40 | 40 | 40 | 40 | 40 | 40 |  |  |  |
| Experimental vignette 2 | Pearson R | .062 | .134 | .333^*^ | .595^**^ | .535^**^ | .320^*^ |  |  |  |
|  | Sig. | .705 | .411 | .036 | .000 | .000 | .044 |  |  |  |
|  | N | 40 | 40 | 40 | 40 | 40 | 40 | 40 |  |  |
| Experimental vignette 3 | Pearson R | .352^*^ | .336^*^ | .276 | .411^**^ | .645^**^ | .475^**^ | .581^**^ |  |  |
|  | Sig. | .026 | .034 | .084 | .008 | .000 | .002 | .000 |  |  |
|  | N | 40 | 40 | 40 | 40 | 40 | 40 | 40 | 40 |  |
| Sexual motivation (Experimental) | Pearson R | -.065 | .208 | .294 | .051 | .248 | .153 | .326^*^ | .133 |  |
|  | Sig. | .689 | .197 | .066 | .753 | .122 | .347 | .040 | .414 |  |
|  | N | 40 | 40 | 40 | 40 | 40 | 40 | 40 | 40 | 40 |
| Trait SOQ (Baseline) | Pearson R | .016 | -.023 | -.088 | -.408^**^ | -.106 | -.215 | -.106 | -.134 | .023 |
|  | Sig. | .923 | .890 | .588 | .009 | .513 | .183 | .517 | .412 | .888 |
|  | N | 40 | 40 | 40 | 40 | 40 | 40 | 40 | 40 | 40 |

*Note.* *. Correlation is significant at the 0.05 level (2-tailed). **. Correlation is significant at the 0.01 level (2-tailed). SOQ = Self-objectification questionnaire.
