# Supplementary material for: In the context of romantic attraction, beautification can increase assertiveness in women
Source: PLoS One. 2020 Mar 10;15(3):e0229162. doi: 10.1371/journal.pone.0229162 (PMC7064170; doi:10.1371/journal.pone.0229162)
Supplement: Attachment — Submitted filename: Response to reviewers 4.docx [file pone.0229162.s002.docx]

**PONE-D-19-16746
Sexually Motivated Beautification Can Increase Assertiveness in Women
PLOS ONE**

**Editor Comments**

**Dear Dr Blake,

Thank you for submitting your manuscript to PLOS ONE. After careful consideration, we feel that it has merit but does not fully meet PLOS ONE’s publication criteria as it currently stands. Therefore, we invite you to submit a revised version of the manuscript that addresses the points raised during the review process.**

**Whilst one reviewer suggested minor revisions, the second reviewer has more substantial concerns and thus recommends a major revision.**

**I enjoyed reading your manuscript, but as I share some of the concerns, I invite you to submit a revised version that addresses the points raised in the reviews.**

Thank you for the opportunity to revise the manuscript for potential publication in PLoS One. Below we respond to your comments, and those of each of the two reviewers.

**Similar to Reviewer 2, one of whose main points concerns the theoretical foundation, I think that a more comprehensive framework will help you reach the main goal of your study stated in the introductory paragraph. In essence, you attempt to show that women’s preoccupation with their physical appearance may not inevitably present a paradox by investigating the conditions under which beautification may increase assertiveness in women. In other words, although it has been shown that it can be a harmful, potentially disempowering pressure on women, beautification may yield positive outcomes in certain circumstances.**

Thank you for this feedback. We have extensively revised the Introduction and General Discussion of the paper to provide a more comprehensive framework for our investigation and effects. In the Introduction of the revised submission, we now explicitly explain two opposing theoretical frameworks for the beauty paradox – one of which predicts that beautification would be associated with low assertiveness (objectification theory), and one of which predicts that beautification would be associated with high assertiveness (sociometer theory). We explain that research on self-objectification and self-sexualization (two related but distinct phenomena to beautification) can inform predictions regarding the effects of beautification on assertiveness, and review this research. We end the introduction by specifying what each theory would predict, and why we might find it theoretically important to control for the confound variables we included in the experiment (pp. 3–9).

In the General Discussion of the revised submission (pp. 25–32), we now begin by re-orienting the reader to the predictions borne from objectification theory and sociometer theory, regarding beautification and assertiveness. We re-emphasize why we measured our confound variables (and their theoretic importance), and explain our results in the context of these two theories. After, we introduce a new section which outlines the implications our results have for understanding related phenomena (such as self-objectification), and for understanding the beauty paradox.

**Given the important contribution such findings might make to current scientific and societal debates, a considerably more careful theoretical treatment of the antecedents, processes, and outcomes of beautification is warranted.**

**For instance, an important question arises in light of Choi and DeLong’s (2019) tenet that “sex appeal” had become so greatly valued in Western society that young women voluntarily imposed sexualisation to the self and of Rollero and De Piccoli’s (2017) findings that personal values (e.g., self-enhancement, self-transcendence, openness to change) influence the degree to which people self-objectify. Notwithstanding a discussion of the relationships between self-objectification and self-sexualisation (see Reviewer 2’s comments on the need of putting your main concepts in perspective), this suggests that person variables beyond trait self-objectification may influence the beautification-assertiveness relationship, but that do not feature in your current theory section.**

Thank you for these recommendations, in the revised submission we have made considerable changes to acknowledge the antecedents, processes, and outcomes of beautification (or where research on these topics does not exist, we have contextualized our understanding by drawing on research on self-objectification or self-sexualization). Much of these changes are in the Introduction (pp. 3–8) and General Discussion (pp. 25–32). We have also incorporated Choi and DeLong (2019) and Rollero and DePiccoli (2017) in the revision. Specifically, we draw on Choi and DeLong (2019) to ensure we present a crisp delineation of the differences between self-objectification, self-sexualization, sexual objectification, and beautification (pp. 3–4, 6). We then acknowledge that one limitation of our findings is that other trait differences relevant to our constructs were not controlled for, and explain the interesting work by Rollero and DePiccoli (2019). Namely, on page 31 we write:

*A final limitation is that we only controlled for one individual difference in our analyses. Although trait self-objectification was a worthy inclusion, many other individual differences affect women’s willingness to beautify, self-objectify, and self-sexualize. Recent work indicates that ideological components related to higher order personal values are especially relevant (Rollero & Piccoli, 2017). Testing whether findings reported here are sensitive to these differences, and the individual differences predictive of beautification, would strengthen our conclusions.*

**Pointing to potential context effects, Yilmaz and Bozo’s (2019) experimental study of women’s state self-objectification, body shame, and negative mood suggests that the processes underlying beautification effects seem to be rather complex. In that study, women’s own look at their body in a swimming costume impacted self-objectification and negative mood significantly more than being looked at by other women or men and there wasn’t a difference between female and male gazes; differences became insignificant, however, for the same women wearing jeans and jumper. Discussing the system of such context effects could help address Reviewer 2’s concerns that you chose an ecologically valid way to assess assertiveness, given that the participants were “compelled to beautify by the experimenters”. I concur that contextual constraints most likely play a substantial part although I wouldn’t see a problem with your experimental manipulation. What assertiveness effects would one expect, however, if beautification were ‘mandatory’, e.g. if stipulated by a women’s employer for an upcoming important client meeting?**

**In sum, a more detailed discussion of the interplay of person and context factors would help specify the conditions that co-determine the potential pressurising and empowering facets of one’s preoccupation with physical appearance and beautification.**

In the revised submission we now raise the important point that contextual effects are likely to be important (pg. 28). Specifically, we write:

*Contextual effects—such as the person a beautified woman believes is judging her (Yilmaz & Bozo, 2019)—are also likely to be important. We focused on beautification in one domain only, and it is unclear whether compelled beautification in other domains (e.g., the workplace) would show similar effects. Likewise, women often become targets of backlash when they act assertively, especially in domains that are stereotype-inconsistent (Rudman & Glick, 2001), and attractive women may be especially likely to be targeted. Women who engage in beautification and appearance-enhancing phenomena can also become targets of aggression by others, men and women like (Arnocky et al., 2019; Blake et al., 2016, 2018; Leenaars, Dane, & Marini, 2008; Vaillancourt & Sharma, 2011). Thus, although increases in beautification may engender benefits to women, in certain contexts it may also engender costs. The contexts under which women may express assertiveness and beautify (and not suffer backlash effects), or the contexts under which women experience beautification as especially disempowering, are important future research topics.*

In response to Reviewer 2’s concerns about ecological validity, we believe this derives from a lack of clarity on our part regarding the why our beautification manipulations occurred in the context of romantic relationships. Although we were careful to state that we aimed only to investigate ‘sexually motivated beautification’ in the original submission, we did not explain our reasons for doing so. In the revision, we explain these reasons. Namely, we explain that sociometer theory predicts that an individual’s self-esteem depends upon the degree to which they are valued as a relational partner, with high self-esteem resulting when people perceive that they have been accepted by others in important domains (page 4). Because attractiveness in the domain of romantic relationships is a domain where women are especially likely to derive self-esteem (Leary & Baumeister, 2000; Meltzer, 2019), we focused on beautification in the context of a potential romantic relationship, as this was the most relevant domain for the theory in question (page 8). Thus, both manipulations asked women to beautify in whatever way was appropriate for them, as if they were entering the context of a potential romantic relationship. This manipulation was ecologically valid for our focus, the problem was that our focus was not clear to the reviewer. We hope that with our extensive revisions, this lack of clarity has been resolved.

**I am confident that you will find both reviewers’ comments helpful in improving your manuscript.

We look forward to receiving your revised manuscript.

Kind regards,

Christian Stamov Roßnagel
Academic Editor
PLOS ONE**

**Journal Requirements:**

**1. When submitting your revision, we need you to address these additional requirements.**

**Please ensure that your manuscript meets PLOS ONE's style requirements, including those for file naming. The PLOS ONE style templates can be found at**
[**http://www.journals.plos.org/plosone/s/file?id=wjVg/PLOSOne_formatting_sample_main_body.pdf**](http://www.journals.plos.org/plosone/s/file?id=wjVg/PLOSOne_formatting_sample_main_body.pdf) **and** [**http://www.journals.plos.org/plosone/s/file?id=ba62/PLOSOne_formatting_sample_title_authors_affiliations.pdf**](http://www.journals.plos.org/plosone/s/file?id=ba62/PLOSOne_formatting_sample_title_authors_affiliations.pdf)

We have amended the manuscript so that it meets PLOS ONE’s formatting requirements, including the title page, tables and figures and the addition of continuous line numbering. We have also changed the referencing to reflect Vancouver Style reference systems.

**2. Please include captions for your Supporting Information files at the end of your manuscript, and update any in-text citations to match accordingly. Please see our Supporting Information guidelines for more information:** [**http://journals.plos.org/plosone/s/supporting-information**](http://journals.plos.org/plosone/s/supporting-information)**.**

Thank you, we have adjusted the S1 Appendix to reflect PLoS style requirements.

**3. We note that several details from the Methods section have been blinded for peer review. Please note that PLOS ONE does not offer double blind peer review. We would be grateful if you could update your Methods section to include all the information previously redacted from the manuscript.
Please note that according to our submission guidelines (**[**http://journals.plos.org/plosone/s/submission-guidelines**](http://journals.plos.org/plosone/s/submission-guidelines)**), outmoded terms and potentially stigmatizing labels should be changed to more current, acceptable terminology. For example: “Caucasian” should be changed to “white” or “of [Western] European descent” (as appropriate).**

We have updated the methods section to include all previously redacted information, and changed the word “Caucasian” to “white”.

**4. Thank you for including your ethics statement:  "The UNSW Ethics Committee approved Study 1 (HREAP 2556) and Study 2 (HREAP 2906). Written consent was obtained for both studies.".**

**a.Please amend your current ethics statement to include the full name of the ethics committee/institutional review board(s) that approved your specific study.**

**b.Once you have amended this/these statement(s) in the Methods section of the manuscript, please add the same text to the “Ethics Statement” field of the submission form (via “Edit Submission”).**

**For additional information about PLOS ONE ethical requirements for human subjects research, please refer to** [**http://journals.plos.org/plosone/s/submission-guidelines#loc-human-subjects-research**](http://journals.plos.org/plosone/s/submission-guidelines#loc-human-subjects-research)**.**

We now specify that UNSW Sydney HREAP stands for The University of New South Wales Sydney Human Research Ethics Advisory Panel (pg 9, pg 21)

**5.  We note that Figure 1 includes an image of participants in the study.**

**As per the PLOS ONE policy (**[**http://journals.plos.org/plosone/s/submission-guidelines#loc-human-subjects-research**](http://journals.plos.org/plosone/s/submission-guidelines#loc-human-subjects-research)**) on papers that include identifying, or potentially identifying, information, the individual(s) or parent(s)/guardian(s) must be informed of the terms of the PLOS open-access (CC-BY) license and provide specific permission for publication of these details under the terms of this license. Please download the** [**Consent Form for Publication in a PLOS Journal**](http://journals.plos.org/plosone/s/file?id=8ce6/plos-consent-form-english.pdf) **(**[**http://journals.plos.org/plosone/s/file?id=8ce6/plos-consent-form-english.pdf**](http://journals.plos.org/plosone/s/file?id=8ce6/plos-consent-form-english.pdf)**). The signed consent form should not be submitted with the manuscript, but should be securely filed in the individual's case notes. Please amend the methods section and ethics statement of the manuscript to explicitly state that the patient/participant has provided consent for publication: “The individual in this manuscript has given written informed consent (as outlined in PLOS consent form) to publish these case details”.**

We are unable to secure these consent forms, so have removed the figure from the revised submission.

**Reviewers' comments:

Reviewer's Responses to Questions

Comments to the Author

1. Is the manuscript technically sound, and do the data support the conclusions?

The manuscript must describe a technically sound piece of scientific research with data that supports the conclusions. Experiments must have been conducted rigorously, with appropriate controls, replication, and sample sizes. The conclusions must be drawn appropriately based on the data presented.

Reviewer #1: Yes

Reviewer #2: Partly**

**2. Has the statistical analysis been performed appropriately and rigorously?

Reviewer #1: Yes

Reviewer #2: I Don't Know**

**3. Have the authors made all data underlying the findings in their manuscript fully available?

The** [**PLOS Data policy**](http://www.plosone.org/static/policies.action#sharing) **requires authors to make all data underlying the findings described in their manuscript fully available without restriction, with rare exception (please refer to the Data Availability Statement in the manuscript PDF file). The data should be provided as part of the manuscript or its supporting information, or deposited to a public repository. For example, in addition to summary statistics, the data points behind means, medians and variance measures should be available. If there are restrictions on publicly sharing data—e.g. participant privacy or use of data from a third party—those must be specified.

Reviewer #1: Yes

Reviewer #2: Yes**

**4. Is the manuscript presented in an intelligible fashion and written in standard English?

PLOS ONE does not copyedit accepted manuscripts, so the language in submitted articles must be clear, correct, and unambiguous. Any typographical or grammatical errors should be corrected at revision, so please note any specific errors here.

Reviewer #1: Yes

Reviewer #2: Yes**

**5. Review Comments to the Author

Please use the space provided to explain your answers to the questions above. You may also include additional comments for the author, including concerns about dual publication, research ethics, or publication ethics. (Please upload your review as an attachment if it exceeds 20,000 characters)**

**Reviewer #1: PONE-D-19-16746

The present work, “Sexually Motivated Beautification Can Increase Assertiveness in Women” addresses the question of whether beautification can empower women to act assertively, doing so via two experiments. I find this research question to be an important one, and, to my knowledge, this work provides some of the first experimental evidence to address the authors’ focal question. Additionally, the work is generally well written, the experimental evidence is somewhat convincing, and the authors take care to not make greater claims than their data can or to sensationalize the claims they do make. I also appreciate the authors’ dedication to open science practices. This work is worthy of eventual publication in PLOS ONE. Below, I make some suggestions for improving the manuscript.**

Thank you for your considered engagement with the manuscript, and for detailing its strengths. Below we address your recommendations for improving it.

**First, let me expand a bit on the important contributions that this work makes to the literature on women and female agency. I see this work as part of a nascent trend in research to question some of the assumptions that many people—especially those of us in liberal-leaning university settings—might hold about women’s behavior. For example, Andrea Meltzer’s recent work (2019, PSPB) challenges whether women are always harmed by being valued for their beauty (as many might assume they are), and Tarran, Muggleton, and Fincher (2019, EHB) challenge whether the suppression of women’s sexually is always enacted by men (as many might assume it is). Here, the authors question whether making oneself more physically attractive can be empowering for women (as many might assume it is not or should not be), and they use an experimental design to explore this. This is paper thus contributes to a larger trend that seeks to describe and understand how women actually think, feel, and behave (and not how many might assume women would or should think, feel, and behave).**

**This paper also engages with some feminist literature, and treats that work and related findings fairly. My main suggestion for improving the work is that the authors lean further into the two sides of the “feminist debate” that they mention on p. 3 in framing their Introduction. There is the possibility to really highlight the diverging perspectives—both with some empirical support—that beautification harms and/or empowers women. The two papers I mentioned above both highlight the two sides of their respective debates very nicely; they could be used as models, especially the former. Focusing the Introduction more the two sides of a debate—which their research question gets to the heart of—will make the paper a more enthralling read for both researchers and lay the public.**

Thank you for suggesting these papers to support the a clearer framing of the Introduction. We were unaware of them and have incorporated them into the revision, and found them very helpful. In the revision, we now explicitly explain two opposing theoretical frameworks for the beauty paradox – one of which predicts that beautification would be associated with low assertiveness (objectification theory), and one of which predicts that beautification would be associated with high assertiveness (sociometer theory; see pp. 3–5 and throughout). We have further explained what each theory would predict, and drawn on both theories to contextualize previous research regarding appearance-enhancing behaviors and assertiveness in women. We hope that the reviewer finds this clearer articulation of our motivating framework enhances the paper, as we do.

**I would also ask that the authors take a bit more text to delineate agency, assertiveness, beautification and (self-)objectification. At first read, we all think that we know what each of these terms means, but they have some useful nuances. A merit of the paper is that is draws all of these terms together, and something as simple as a parenthetical definition—perhaps even from the work they are citing at the moment (e.g., Liss et al., 2011 on self-objectification)—would do.**

In the revised introduction, we have taken care to carefully delineate agency from assertiveness, and self-objectification from beautification, sexual objectification, and self-sexualization (pp. 3–4, 6). We now articulate and define beautification, self-objectification, sexual objectification, and self-sexualization explicitly. We have further characterized how findings regarding self-sexualization and self-objectification may provide insight into beautification and agency.

Regarding agency and assertiveness, in the original submission we did define agency and explain that assertiveness was a manifestation of agency. However, in the revision we have made this clearer, and added specific hypotheses regarding what each theoretical framework predicts regarding how beautification may affect assertiveness (pp. 5–8).

**I also share some more minor suggestions. Most of these suggestions are to add further nuance and/or further speculation as to the causes and consequences of the effect.

- It would be beneficial if the authors would speculate a bit more as to how and why beautification might have this effect. That is, what is the proximate and/or ultimate logic? For example, for women, beauty might be considered a source of power for reasons consistent with ultimate logic (a la Sell, Tooby, & Cosmides, 2009). This might also be a place to mention Leary’s (2005) sociometer work; that is, perhaps the effect lies in the fact that other people treat more attractive women more favorably—and/or women expect that other people will do so—which could influence women’s assertiveness.**

Thank you for the suggestion. In the introduction, we have now used sociometer theory as a framework to explain the positive effect of beautification on assertiveness (pp. 3–5).

**- Related, I’m wondering whether it is the act of beautification, the feeling of having become more beautiful, and/or the expectation that others will find them more beautiful that is perhaps driving this effect.**

We wonder the same thing. In our data, we hoped by including public and private assertiveness tasks in Experiment 2, we might gain some insight into whether tasks that involved being seen by others resulted in more assertiveness, compared with those that were private tasks. Unfortunately we did not find clear differences between the two tasks, so we are unable to shed light on this issue. We’ve noted this in the original submission in the discussion (page 26).

**- People might often assume that this effect is solely positive for women. (They might immediately think: A step toward gender equality maybe? Yay!). But increased beautification is likely to engender not only possible benefits (assertiveness), but also possible costs. Indeed, the authors discuss this in the GD (e.g., p. 24-25). There are additional costs they might note. For example, assertiveness might also have some costs, especially for women. Moreover, women who engage in beautification might also receive more aggression from other women (e.g.,** **Leenaars, Dane, & Marini, 2008; Vaillancourt & Sharma, 2011).

- Related, for the discussion of beauty and agency on p. 26-27, see also Arnocky et al.’s (2019) recent Psychological Science paper.**

Thank you, we have added this paragraph to the discussion (page 28):

*... Likewise, women often become targets of backlash when they act assertively, especially in domains that are stereotype-inconsistent (Rudman & Glick, 2001), and attractive women may be especially likely to be targeted. Women who engage in beautification and appearance-enhancing phenomena can also become targets of aggression by others, men and women like (Arnocky et al., 2019; Blake et al., 2016, 2018; Leenaars, Dane, & Marini, 2008; Vaillancourt & Sharma, 2011). Thus, although increases in beautification may engender benefits to women, in certain contexts it may also engender costs. The contexts under which women may express assertiveness and beautify (and not suffer backlash effects), or the contexts under which women experience beautification as especially disempowering, are important future research topics.*

**- I would have expected the assertiveness to be a bit more domain-specific, mating-related, and primarily confined to less formal interactions with men. Some of the DVs from Experiment 2 (vignettes) and the use of a more formal interaction (i.e., the mock job interview) might begin to probe this a bit. (I’ll confess that I did not think the vignettes were the most solid operationalizations of private/public.) Then again, beauty might cue benefits beyond mating (e.g., Eisenbruch, Lukaszewski, & Roney, 2017), so perhaps it might also increase assertiveness beyond mating domains. I think some speculation regarding women’s audience(s) and to whom/in which situations this assertiveness might be most expected would be useful. (For the job interview, please provide a bit more information in the main manuscript. E.g., Was the interviewer male, an experimenter who simply gave the P the sheet, …?)**

As noted, we hoped to provide insight into the domains most likely to be affected by beautification-induced assertiveness by investigating domains that were related and unrelated to appearance. Unfortunately, our results were inconclusive on this front. In the discussion, we explain this limitation of our results, and our inability to draw conclusions about whether assertiveness effects were domain-specific or domain-general (page 26, page 30). We further note that based on sociometer theory, our speculation would be that beautification-induced assertiveness would be strongest in appearance-related domains, where valuations are highly relevant to women’s self-esteem, and weaker, albeit present in other domains (page 30). Future investigations into this speculation would be a welcome addition to future work.

**- As the author of a number of papers that focus on women’s psychology and behavior, I won’t require the authors here to speculate as to how this same process might play out among men; men were not their focus and for theoretically-sound reasons. (Still, in the West, men have increasingly become objects of the human gaze, and this might have serious practical implications for approximately 50% of the population.)**

Thank you for this understanding.

**- One final minor comment (not minor suggestion): This paper, and the feminist debate with which it engages, calls to mind an interesting problem. Beautification might make women feel bad about themselves, might diminish women, might require women to spend finite time and money on hair products (meaning that time and money cannot be spent on “better” pursuits). But beautification might also make women more assertive in some instances. But then again, this increased assertiveness might owe to the fact that women live in a society where they are valued for their beauty, so is this “authentic” assertiveness? (The authors deal with this briefly on p. 5.) Who gets to say what is “authentic” assertiveness that “genuinely” empowers women? Do scholars? Do women themselves? Again, the experimental, empirical aspect of this paper provides meaningful contribution, in part by cutting through this.**

We understand your sentiment, and this rationale was the motivating reason for the study. The dominant framework in this issue informs us that beautification is disempowering, yet many women report that it is empowering. Thus, who is ‘correct’? As you note, we hoped to cut through this conundrum with experimental investigation, and we hope our work sheds some light on this important topic.

**Reviewer #2:**

**Research reveals links between women’s beautification and a variety of outcomes, both positive and negative. The current work extends this empirical literature by testing for effects of women’s beautification on their assertive attitudes, behaviors, and intentions. Despite notable strengths of this work related to its aim and experimental rigor, I have concerns related to the theoretical foundation, how key constructs were operationalized, and potential factors that covary with the beautification condition. I detail these concerns and other questions, and provide suggestions that I hope will be helpful, below.

1. The authors frame this work as addressing a paradox related to women’s beautification efforts. Although this is a question of potential practical importance, the authors do not present a theoretical framework to guide the statistical tests presented within the manuscript. As is, it is not clear that the current tests were designed with resolving this paradox in mind. (For example, boundary conditions related to the potential positive vs. negative effects of these efforts are not considered). An organizational theoretical framework, or the presentation of competing theoretical frameworks, would bring clarity and structure to this work. An explicit theoretical framework might also address questions related to the selected measures and instruction sets, as described below.**

Thank you for this considered engagement with the submission. We can understand the reviewer’s need for a more explicit specification of a guiding theoretical framework, and agree that this would enhance the paper. We have now extensively revised the Introduction to provide this framework (pp. 3–8). There we provide a background of two opposing schools of thought regarding the beautification paradox – one of which predicts that beautification would be associated with low assertiveness (objectification theory), and one of which predicts that beautification would be associated with high assertiveness (sociometer theory). We also ensure we have clearly delineated beautification, self-objectification, self-sexualization, and sexual objectification, for a crisper theoretic framework. We explain that research on self-objectification and self-sexualization (two related but distinct phenomena to beautification) can inform predictions regarding the effects of beautification on assertiveness, and review this research. We end the introduction by specifying what each theory would predict, and why we might find it theoretically important to control for the confound variables we included in the experiment (pp. 3–9).

In the General Discussion of the revised submission (pp. 25–32), we now begin by re-orienting the reader to the predictions borne from objectification theory and sociometer theory, regarding beautification and assertiveness. We re-emphasize why we measured our confound variables (and their theoretic importance), and explain our results in the context of these two theories. After, we introduce a new section which outlines the implications our results have for understanding related phenomena (such as self-objectification), and for understanding the beauty paradox.

We hope that the reviewer finds this clearer articulation of our motivating framework enhances the paper, as we do.

**In sum, the theoretical (and practical, see below) implications of this work should be more clearly elucidated. Without a clear theoretical framework, it is difficult to discern the importance of the present analyses. (For instance, analyses pertaining to individual differences in sexual motivation were presented as exploratory in Experiment 1, but become a focal analysis in Experiment 2. The theoretical and/or practical reason(s) for this change in perspective is not articulated.) This work would benefit from a more explicit specification of the guiding theoretical framework(s), and a clear delineation of which results would support (or refute) this view.**

As noted above, we have now more clearly articulated the motivating framework for the paper in the Introduction and General discussion.

Our reasons for examining sexual motivation specifically were explained in the introduction in the original submission. There, we noted that theorists argue that some forms of beautification elevates agency because they are sexually empowering (Lerum & Dworkin, 2009). We explained that this raises the possibility that beautification will only confer elevated agency to the extent that it increases women’s sexual motivation. We then explained that we looked at the relationship between sexual motivation, beautification, and assertiveness in an exploratory fashion in Experiment 1, then sought to confirm relationships between variables in Experiment 2. In the revised submission, we have aimed to make this clearer, by explaining this reasoning in the section entitled “The Current Experiments”. There we explain:

*We also examined the relationship between beautification and assertiveness, controlling for sexual motivation.* *Theorists argue that some forms of beautification elevate agency because they are sexually empowering (Lerum & Dworkin, 2009). This idea, which is consistent with state assertiveness positively correlating with state sexual motivation in women (Blake, Bastian, O'Dean, & Denson, 2017), raises the possibility that beautification will only confer elevated agency to the extent that it increases women’s sexual motivation. To see if the effect of beautification on assertiveness was due to increased sexual motivation, in Experiment 1 we examined the relationship between these variables in an exploratory fashion. In Experiment 2, we sought to reproduce and extend statistical associations from Experiment 1. Finally, to control for relevant individual level confounds, in Experiment 2 we examined and controlled for trait self-objectification. Scholars have noted a considerable conceptual murkiness between self-objectification and related phenomena (Choi & DeLong, 2019), and we aimed to control for trait differences in the tendency to self-objectify to provide a crisper dissection of resulting effects.*

**2. The conceptual overlap between measured constructs, as well as the interchangeable use of labels made it difficult to discern some of what the authors were attempting to assess and test. This broad concern was reflected in construct operationalization, measurement, and interpretation. For instance:

The authors use the terms “sexualization” and “beautification” nearly interchangeably throughout the manuscript. It is unclear if their primary hypotheses concern beautification effort or sexualization of one’s appearance (or perhaps sexual motivation). One might make different predictions regarding the potential effects of beautification versus sexualization on assertiveness (as well as the boundary conditions, moderating and mediating factors). This seems like a critical distinction to make, given this was the primary independent variable in the current work.**

In the original submission, we explained that our focus was on sexually motivated beautification specifically –in other words, beautification in the context of romantic relationships. For this reason, we our use of terms was somewhat muddied, and we did, at times, conflate beautification and sexualization. Partly this was driven on their being little work specifically on beautification, but much research on self-objectification and self-sexualization. In the revised submission, we have corrected this issue, and taken care to specify that when we mention literature regarding self-sexualization or self-objectification, we do so to explain how related research can inform the current work. We have also added a new section to the introduction that explains our theoretical reason for focusing on beautification in the context of romantic attraction specifically (page 4), and updated the title and abstract to make this clearer. Further, in the introduction, we now explicitly delineate self-objectification, self-sexualization, sexual objectification, and beautification, to aid clarity of interpretation, measurement, and operationalization (pp. 3–4, 6).

**The authors also use the words agency and assertiveness in overlapping ways. It would be helpful to clearly operationalize the key outcome of interest in this work. Related to this point, it seems that the measure of positive affect used in Experiment 1 contains items that substantially overlap with feelings of assertiveness (e.g., confident, active, fearless, daring, strong, attentive, bold, lively, determined; NOT timid or afraid). It seems important to clearly define and operationalize key constructs, use consistent labels when referring to these constructs, and avoid overlap in the measurement of these constructs.**

In the original submission we provided a review of the concept of agency, and explained that assertiveness is a form of agency. Thus, we do sometimes use these words interchangeably, as we have specified that one falls under the umbrella domain of the other. However, we can see that some further precision is warranted. To accommodate the reviewer’s comment, in the revised submission we have corrected any improper use of the word ‘agency’, if ‘assertiveness’ was more appropriate. We have further taken the liberty of explaining why the focus on assertiveness was an appropriate one to answer our research question (pp. 5–8).

We used the general positive affect scale from the PANAS-X by Watson and Clark to measure positive affect. The PANAX is the most widely used and validated measure of affect currently known, with over 33K citations. The general positive affect scale comprised of the following items: “active, alert, attentive, determined, enthusiastic, excited, inspired, interested, proud, strong”. Although some of these items are related to agency, changing the calculation of the scale would reduce its validity. That our results are robust to positive affect, especially given that positive affect contains some concepts related to agency, actually strengthens the results of our analysis. It indicates that they are robust even to controlling for moods that are related to the agency concept.

**Finally, and related to Point #1, I wondered why the “behavioral intentions” assertiveness measure included in Experiment 2 focused exclusively on consumer domains. Assertive behavior, it is reasonable to assume, could be assessed across many domains. (That is, there seems to be no theoretical rationale for predicting that the enhanced assertiveness should only be expressed in consumer domains.) Further, it is unclear why the distinction between public vs. private, and appearance-related vs. not, was important to assess here.**

Our rationale for extending the work to consumer domains was stated in the original submission in the introduction of Experiment 2, and discussed in the General Discussion. We explained that we hoped to determine whether beautification increased assertiveness in appearance-related as well as appearance-unrelated domains, but ultimately the findings were inconclusive (page 19, page 26). We have re-written the General Discussion to try to make this more apparent. The public versus private dichotomy was included because is important in consumer behaviour. We did not specify this in the original submission, so have added this to the revision (page 19).

**3. I also had concerns and questions related to the instructional sets presented to participants (and subsequent participant exclusions) and potential confounds related to post-experiment plans.

Regarding the instruction sets, it is unclear why participants in the beautification/sexualization condition were prompted to ready themselves for a “hot date.” This seems likely to have primed mating motivations among most participants. This also seems methodologically-inappropriate if this work was intended to test the effects of beautification per se (rather than sexualization or mating motives) on assertiveness. Beautification efforts can certainly take place in the absence of explicit mating motivations. Related to this point, why were participants excluded based on non-heterosexual orientation? There is no explicit theoretical or empirical rationale provided for the relevance of women’s sexual orientation in understanding the link between beautification and assertiveness. (Non-heterosexual women also engage in appearance enhancement.) Further, I am unsure if this is an ecologically valid way to assess potential empowerment (or assertiveness) effects that might derive from women choosing to beautify, given that the female participants were compelled to beautify by the experimenters.**

In the revised submission, we now explain why we focused on beautification in the context of romantic relationships. Namely, we state: “Because attractiveness in the domain of romantic relationships is a domain where women are especially likely to derive self-esteem (Leary & Baumeister, 2000; Meltzer, 2019), we focused on beautification in the context of a potential romantic relationship (page 8).” Focusing specifically on beautification in one context was always an aim of the current work, but one that perhaps could have been better explained.

We excluded two women who were homosexual because lesbian beauty norms differ from heterosexual beauty norms (Myers, Taub, Morris, & Rothblum, 1999), and we aimed to reduce this source of variance in the study. We can confirm, however, that including these two women largely replicated the previously reported findings.

**Across Experiments 1 and 2, I wondered if participants were told that they would be expected to return to their baseline state of attractiveness (e.g., “unbeautify”) before leaving (or ending the session). If participants were not required to do this, any effects that took place during the experimental session could be due to differences in plans among participants who engaged in beautification efforts vs. did not. It is reasonable to assume that participants who left the experiment wearing the outfits displayed in Figure 1, Panel A, would be more likely than the control participants to seek out social interactions with a variety of partners after the session (e.g., after investing this effort, they might have decided they would actually meet up with someone for a “hot date”). From this view, one could interpret any between groups differences as deriving from the thoughts, feelings, and expectations of women’s post-session plans. Of course, there is no way to know this was the case. However, this possibility cannot be ruled out if participants were not instructed to return to their baseline state.**

Participants were told they could return to their baseline level of attractiveness at the end of the both experiments. In Experiment 1, where participants did the experiment at the laboratory, we also provided makeup wipes and other materials to help women remove their makeup, should they wish to, and provided them private changerooms to change outfits.

**4. Experiment 2 was presented as a replication of Experiment 1. However, there were important differences between these two experiments (beyond the within- vs. between-person design). First, as noted above, the analyses in Experiment 2 focused heavily on sexual motivation and self-objectification as moderators of the relationship between beautification and the varied measures of assertiveness. However, it is unclear why these moderators became a central focus. A clear theoretical rationale for these tests was not provided. Further, the analyses related to sexual motivation were presented as exploratory in Experiment 1 but were then emphasized in Experiment 2 based on the significant mediation analysis in Experiment 1, even though (again), a clear rationale for why this variable was important (other than the significant finding) was not presented. Finally, sexual motivation was tested as a mediator in Experiment 1 and a moderator in Experiment 2. If the point was to replicate Experiment 1, why wasn’t sexual motivation tested as mediating the relationships between beautification and assertiveness? (That is, sexual motivation should have been assessed at both timepoints, with the difference score tested as a mediator in Experiment 2.)**

Experiment 2 was not designed to directly replicate Experiment 1, but instead as a conceptual replication. It is for this reason that there were differences between the two experiments. In the introduction in the original submission, we explained that Experiment 2 differed from Experiment 1 by using a within-subject design, and by controlling for trait self-objectification. We explained that controlling for self-objectification would allow us to account for relevant confounds (in the revised submission, we now clarify that these confounds were trait level, individual confounds, p. 9).

We focused on sexual motivation as a moderator because some theorists argue that some forms of beautification elevate agency only because they are sexually empowering. As stated in the introduction in the original submission, in Experiment 1, we tested these relationships in an exploratory manner, but we aimed to confirm them in Experiment 2. We did not include self-objectification or sexual motivation as mediators in Experiment 2 because they were not measured at both time points, thus, statistically, we were unable to do so.

**5. In addition to the dubious theoretical implications of this work, the practical implications could be debated. One might assume that assertive attitudes are practically important insofar as they motivate assertive behavior. Experiment 1 failed to find a direct effect of beautification on assertive behavior, and Experiment 2 implications were limited by an exclusive focus on consumer assertiveness.**

Thank you, we extensively note this limitation throughout the original submission (p. 1, 26, 30).

**6. In the abstract, the authors state that this work “provides important, novel insight into motivations that underlie women’s appearance-enhancing behaviors.” The current work does not provide much insight into the motivations underlying women’s attractiveness enhancement, given that women were explicitly instructed to beautify for a hot date. (That is, there is no variation in their motivation to self-beautify given that they all beautified in response to experimental instructions.)**

We have corrected the sentence to read: “These experiments provide an important, novel insight into the effects of women’s appearance-enhancing behaviors, showing that beautification may positively affect women under some circumstances (page 1).”

**7. Based on cell sizes, it appears that participant exclusions were not randomly distributed across conditions (i.e., there were fewer participants in the beautification condition in Experiment 1). Have the authors considered if (and how) this could have affected their results? For instance, is it possible that less assertive participants were less able or willing to follow the instructions in the beautification condition?**

In the original submission we explained in footnote 1 that the online pre-screening survey randomly allocated 113 women into the control condition and 106 women into the sexualized clothing condition. Differences in participation between conditions were non-significant, χ^2^ (1, *N* = 219) 0.83, *p* = .363.
